# Delayed emergence from anesthesia caused by an intraoperative cerebral embolism of a malignant peripheral nerve sheath tumor in a neurofibromatosis type 1 patient: a case report

**DOI:** 10.1186/s40981-023-00614-y

**Published:** 2023-05-11

**Authors:** Keishi Kawano, Makiko Tani, Hiroshi Morimatsu

**Affiliations:** 1 Department of Anesthesiology, Okayama City Hospital, 3-20-1, Kitanagase-Omotemachi, Kita-Ku, Okayama, 700-8557 Japan; 2 Department of Anesthesiology and Resuscitology, Graduate School of Medicine Dentistry and Pharmaceutical Sciences, Okayama University Hospital, 2-5-1, Shikata-Cho, Kita-Ku, Okayama, 700-8558 Japan

**Keywords:** Delayed emergence, Cerebral tumor embolism, Endovascular thrombectomy, Neurofibromatosis type 1, Malignant peripheral nerve sheath tumor, Lung surgery

## Abstract

**Background:**

Malignant peripheral nerve sheath tumors (MPNSTs) are aggressive soft tissue sarcomas which commonly arise from neurofibromatosis type 1. Lung metastases of the tumors are well-known, but intraoperative cerebral tumor embolisms of MPNSTs have not been reported in literature.

**Case presentation:**

A 52-year-old female patient with neurofibromatosis type 1 underwent a right lung partial resection for lung tumors. She was extubated after adequate recovery of spontaneous breathing; however, she could not respond to verbal commands. In the intensive care unit, her neurological examination revealed conjugate eye deviation, right hemiparalysis, and aphasia. Magnetic resonance imaging revealed acute cerebral ischemia, so she underwent an endovascular thrombectomy. The histopathological diagnosis of emboli was a MPNST, which was identical with that of the resected lung tumor.

**Conclusion:**

We report the first case of delayed emergence caused by a cerebral tumor embolism of MPNST during partial lung resection.

## Background

A malignant peripheral nerve sheath tumor (MPNST) is associated with neurofibromatosis type 1 (NF-1). NF-1 is known as an inherited disease which is characterized by café au lait spots and development of tumors in the central and peripheral nervous systems. NF-1 is also associated with other organ tumors, such as optic nerve glioma, gastrointestinal stromal tumors, and breast tumors. MPNST is an aggressive sarcoma, and 8–13% of NF-1 patients are reported to develop MPNST [1, 2].

MPNSTs occur commonly in the trunk, extremities, head, and neck [3], and over 50% of metastases occur in lungs, followed by intra-abdominal viscera and bone [4]. To the contrary, brain metastasis is unusual [5]. To our best knowledge, cases of cerebral arterial embolism of MPNST, particularly occurring intraoperatively, have not been reported in literature. Here, we present a case that was complicated by delayed emergence from general anesthesia due to an intraoperative cerebral artery embolism of MPNST.

## Case presentation

The patient was a 52-year-old female with well-controlled hypertension. Two years ago, she had undergone a mastectomy for a breast tumor, which was pathologically diagnosed as MPNST associated with NF-1. In a chest computed tomography (CT) follow-up scan for the breast tumor, multiple lung tumors were noted. Although there was no enhanced CT scan, neither intracardiac mass nor intrapulmonary vein mass was detected. The brain was out of the scan range in this CT. She was then scheduled to undergo a video-assisted right lung partial resection for the lung tumors. She had no past medical history of cardiac dysfunction or arrhythmias. Preoperative physical examination was normal. She showed no abnormal neurological findings and performed normal activities of daily living. Preoperative laboratory data were unremarkable.

**Fig. 1:**
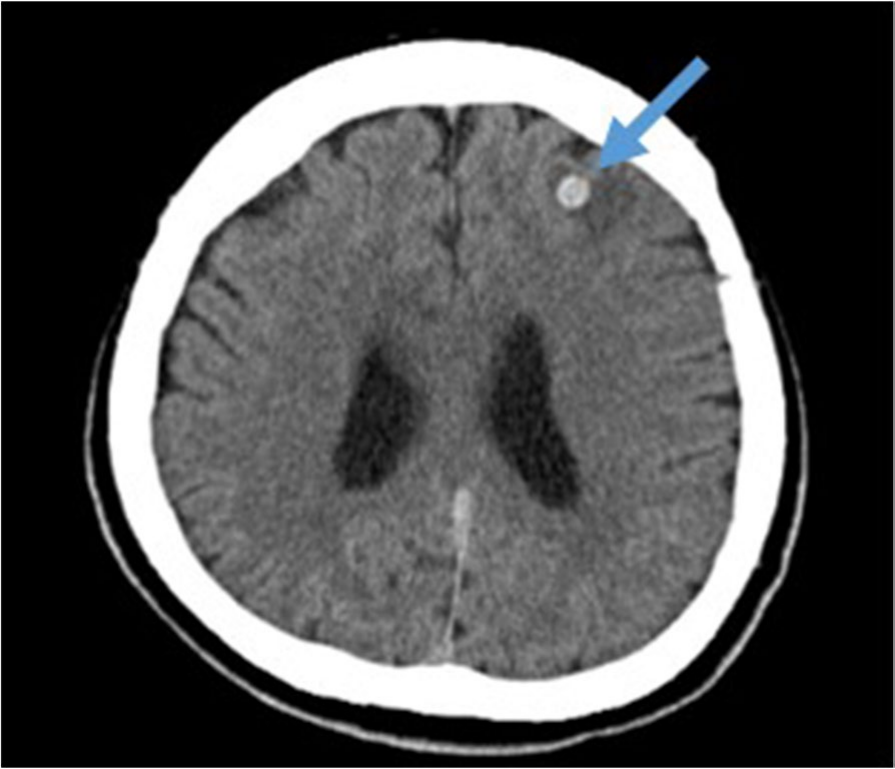
Postoperative brain CT scan. Postoperative brain CT scan 2 h after extubation shows only a high-density lesion surrounded by a low-density area in the left frontal lobe (blue arrow). The lesion was presumed to be old bleeding before further investigation. CT, computed tomography

**Fig. 2:**
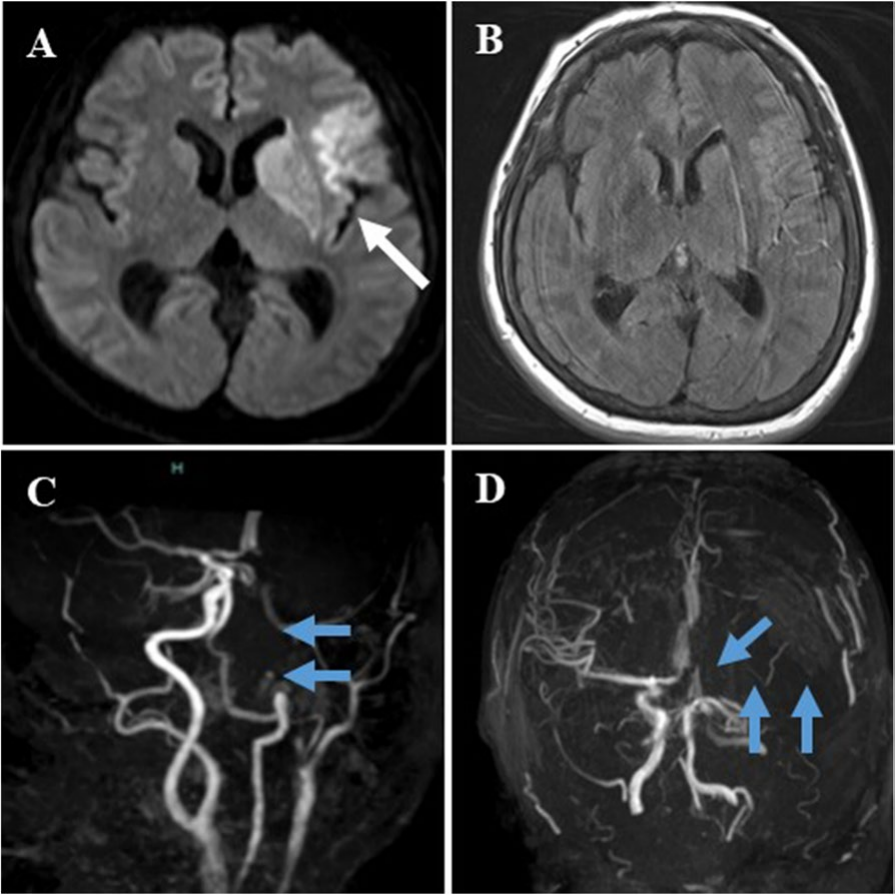
Postoperative MRI. **A** High-intensity area of patient’s left frontal lobe in the diffusion-weighted image (white arrow). **B** No intensity changes of her left frontal lobe in the fluid-attenuated inversion recovery image. **C** and **D** MR angiography showing total occlusion of her left internal carotid artery (blue arrows). MRI, magnetic resonance imaging

**Fig. 3:**
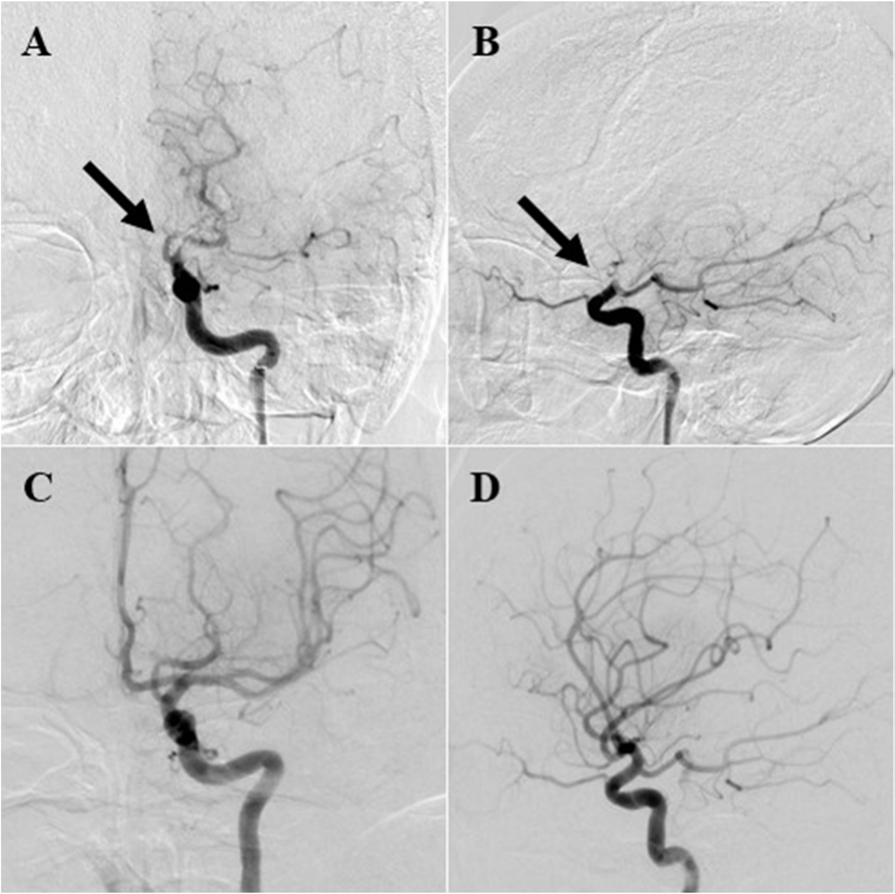
Angiography images. Left internal carotid artery occlusion (black arrows). **A** Coronal view. **B** Lateral view. Endovascular thrombectomy was successfully achieved. **C** Coronal view. **D** Lateral view

On the day of the surgery, she walked on her own into the operating room. General anesthesia was induced with propofol, remifentanil, and rocuronium, and her trachea was intubated with a double-lumen tube. Anesthesia was maintained at 2.5 to 3 mcg/ml with effect site concentration of propofol predicted by a target control infusion (TCI) device and continuous infusion of remifentanil at 0.05 to 0.23 mcg/kg/min. Her mean arterial pressure was maintained between 65 and 100 mmHg without significant hemodynamic changes. Atropine was administered during induction due to bradycardia, but no other vasoactive agonists were necessary afterwards. The patient state index (PSI) showed between 30 and 55 under SedLine™ (Masimo Corporation, Irvine, CA, USA) monitoring without any specific findings of electroencephalography. Total dose of fentanyl during anesthesia was 200 mcg. At the conclusion of the surgery, 20 ml of 0.375% ropivacaine was injected as an intercostal nerve block. The surgery was completed in 68 min, and we discontinued intravenous anesthetics and administered sugammadex. We confirmed that train of four ratios was over 90%, and that effect site concentration of propofol was below 1 mcg/ml. She could not respond to verbal commands or move her limbs, but she opened her eyes by verbal stimuli, and her minute volume was sufficient by spontaneous ventilation. We then extubated her trachea, noting that it was 3 h after the anesthesia induction. The patient was transferred to the intensive care unit (ICU) for further observation. In the ICU, we excluded hypercapnia, hypo/hyperglycemia, hypo/hypernatremia, and hypothermia as causes of impaired consciousness. She was still drowsy for 2 h after the extubation, and her neurological examination revealed conjugate eye deviation to the left, right hemiparesis, and aphasia. We suspected a stroke and performed a brain CT scan which revealed a small size of high-density area surrounded by low density in her left frontal lobe (Fig. 1). However, her symptoms were not explainable with this finding, and we thought another lesion must have existed. Subsequent magnetic resonance imaging (MRI) indicated a high-intensity area of her left frontal lobe in the diffusion-weighted image (Fig. 2A), whereas the fluid-attenuated inversion recovery image of the MRI showed no high-intensity area (Fig. 2B). These findings indicated acute cerebral ischemia. In addition, MR angiography demonstrated total occlusion of her left internal carotid artery (Fig. 2C and D). She was directly transferred to the angiography suite, and she underwent endovascular thrombectomy under monitored anesthesia care with continuous administration of dexmedetomidine and local anesthesia without any complications such as hypoxemia due to upper airway obstruction or hemodynamic instability (Fig. 3). The largest aspirated embolus was approximately 3-cm length. Immediately post-thrombectomy, she began to pull her limbs away from painful stimulus. At this point, we listed three other differential diagnoses for her acute embolism: Trousseau’s syndrome (also known as cancer-associated thrombosis), cardiogenic thrombosis, and paradoxical thrombosis. We then checked D-dimer and performed an electrocardiogram echocardiography. D-dimer was not high, and there was no evidence of perioperative arrhythmia in the electrocardiogram throughout the perioperative period, intracardiac mass, or right-left shunt in the echocardiography. In addition, the removed emboli were white, which suggested that the emboli consisted of tumors and not blood.

On postoperative day (POD) 1, we administered edaravone infusion without anticoagulants due to the possibility of intracranial bleeding. On POD 6, her follow-up CT scan demonstrated slight intracranial bleeding. We did not induce antithrombotic therapy as a prevention for systemic embolism. Her hemiplegia and aphasia gradually improved through daily rehabilitation, and she was transferred to the ward on POD 7. Consequently, on POD 15, pathological diagnosis revealed that the emboli were MPNST, the same pathology of the lung tumor and the breast mass previously resected.

## Discussion

Delayed emergence is a state of inadequate level of consciousness 30–60 min after the end of general anesthesia [6] due to various causes. Neurological complications such as intraoperative cerebral hypoxia, hemorrhage, or thrombosis are included in those causes [6].

Incidence of perioperative acute ischemic stroke in lobectomy or segmental lung resection was reported to be 0.6% [7]. Perioperative ischemic stroke is important and requires prompt diagnosis and treatment. Otherwise, the condition elevates perioperative morbidity and mortality. For acute ischemic stroke caused by acute occlusion of the internal carotid artery, mechanical thrombectomy is strongly recommended [8] within 24 h if recombinant tissue-plasminogen activator (rt-PA) is contraindicated. A meta-analysis provided evidence regarding association of shorter time from symptom onset to actual reperfusion with better outcomes among patients who underwent endovascular thrombectomy [9]. We monitored the SedLine™ during the surgery, but our retrospective review of electroencephalography revealed no remarkable changes which could indicate cerebral ischemia. Thus, we found it difficult to identify the exact onset of the ischemic stroke. As she showed no neurological deficits before induction of the surgery, the onset of the ischemic stroke was estimated within 4.5–6.5 h, and rt-PA was contraindicated due to recent surgery. Therefore, we decided to perform a mechanical embolectomy. Reperfusion was obtained in 5.5–7.5 h after the onset of the stroke. Our immediate workup led us to an accurate diagnosis and good outcome after acute cerebral ischemia.

Anesthesia strategy during endovascular thrombectomy can be largely divided into general anesthesia and local anesthesia. General anesthesia can secure opening of airway and immobilization of patients, whereas local anesthesia has advantages including shorter time of induction for the procedure and lower tendency of hypotension, which is frequently observed under general anesthesia [10, 11]. Brinjikji et al. published a meta-analysis comparing outcomes of patients who undergone endovascular stroke revascularization under general anesthesia with those receiving either local anesthesia or conscious sedation [11]. They reported that patients under general anesthesia had higher odds of 90-day mortality, respiratory complications, and lower functional outcomes. Based on this evidence, we selected monitored anesthesia care with dexmedetomidine and local anesthesia, and the procedure finished uneventfully.

To the extent of our knowledge, there have been no cases reported as an MPNST embolizing cerebral artery. This is probably the first case report of MPNST causing embolic acute cerebral ischemia during lung surgery.

Cerebral artery tumor embolism induces acute neurological disorder; however, it is rarely diagnosed because of its low incidence and necessity of histological confirmation [12, 13]. There is one published review of histological diagnoses of 14 cases which had undergone endovascular mechanical embolectomy for tumors causing acute cerebral ischemic stroke [14]. According to the review, only two of 14 were diagnosed with sarcoma [14]. In our case, the emboli were histologically uncommon because MPNST is a sarcoma that accounts for only 2–4% of all soft tissue sarcomas [15].

Regarding the surgery-related mechanism causing the arterial tumor embolism of MPNST in our case, there are two possibilities: (1) lung metastasis of MPNST invading the pulmonary veins and (2) intracardiac MPNST. The patient did not undergo transthoracic echocardiography preoperatively, and there was no definite evidence of tumor invasion into the pulmonary veins. However, lung cancer is reported to commonly invade pulmonary veins [16]. In addition, Prioleau et al. reported that surgical handling during dissection is a major factor of perioperative malignant tumor embolism [17]. In our case, there is a possibility that minor invasions of the lung tumor into the pulmonary veins resulted in arterial tumor embolization during the surgical procedure, which were undetectable in preoperative CT scan. Cardiac metastasis of MPNST is rare but is mostly preceded by lung metastasis [18]. We did not find evidence of cardiac involvement of the tumor by postoperative echocardiography; however, in this case of lung metastasis of MPNST, there was a possibility of small cardiac involvement of MPNST, and that became the emboli in the cerebral artery.

While brain metastases of MPNSTs have rarely been reported in literature [5], Park et al. reported that there were three previous cases of MPNSTs which presented as hemorrhagic cerebral metastasis preceded by lung metastasis [19]. Accordingly, we should take a possibility of cerebral metastasis into consideration on perioperative management of MPNST patients with lung metastases. We retrospectively reviewed in our case that the CT scan shown in Fig. 2 could demonstrate a lesion of brain metastasis. When we encounter delayed emergence from anesthesia in an MPNST patient, regardless of surgical handling on the main pulmonary veins during lung surgery, it is essential that we perform prompt MRI with a suspicion of intraoperative cerebral tumor embolization as a cause of delayed emergence, even after non-lung surgery as well.

## Conclusion

We presented the first case of a cerebral tumor embolism of MPNST during lung surgery which required endovascular embolectomy. The cerebral embolism presented as a delayed emergence from general anesthesia. An immediate neurological examination provided an accurate diagnosis followed up by appropriate treatment.

## Data Availability

Not applicable due to patient privacy concerns.

## Abbreviations

MPNST: Malignant peripheral nerve sheath tumor
NF-1: Neurofibromatosis type 1
CT: Computed tomography
TCI: Target control infusion
PSI: Patient state index
ICU: Intensive care unit
MRI: Magnetic resonance imaging
POD: Postoperative day
rt-PA: Recombinant tissue-plasminogen activator
